# Mutational Analysis Supports Three-Hairpin Model of Attenuator for Transcription Regulation of *ilvBN*C Operon in *Corynebacterium glutamicum*

**DOI:** 10.3390/microorganisms13020291

**Published:** 2025-01-28

**Authors:** Ludmila E. Ryabchenko, Igor I. Titov, Tatyana E. Leonova, Tatyana I. Kalinina, Tatyana V. Gerasimova, Marina E. Sheremetieva, Nikolay A. Kolchanov, Tamara M. Khlebodarova, Alexander S. Yanenko

**Affiliations:** 1 National Research Center “Kurchatov Institute”, Kurchatov Genomic Center, Akademika Kurchatova pl. 1, 123182 Moscow, Russiayanenko_as@nrcki.ru (A.S.Y.); 2 Department of Systems Biology, Institute of Cytology and Genetics SB RAS, Akademika Lavrentyev Ave., 10, 630090 Novosibirsk, Russia; titov@bionet.nsc.ru (I.I.T.); tamara@bionet.nsc.ru (T.M.K.); 3 Kurchatov Genomic Center, Institute of Cytology and Genetics SB RAS, Akademika Lavrentyev Ave., 10, 630090 Novosibirsk, Russia

**Keywords:** *Corynebacterium glutamicum*, transcription regulation, attenuation, *ilvBNC* operon, acetohydroxy acid synthase (AHAS)

## Abstract

The *ilvBNC* operon in *Corynebacterium glutamicum* encodes key enzymes for the biosynthesis of branched-chain amino acids (L-isoleucine, L-leucine, and L-valine). This operon has been studied for quite a long time, and it is assumed that three hairpin mRNA structures can be formed in its regulatory region; however, their functionality and role in the attenuation mechanism of the *ilvBNC* operon are not completely clear. In the present work, we performed a mutational analysis of mRNA secondary structures in the regulatory region of the *C. glutamicum ilvBNC* operon, which allowed us to propose a model of the regulation of its transcription involving three mRNA hairpins that essentially act as a transcription terminator, an antiterminator, and an antiantiterminator. In this work, we proved the existence of a transcription terminator in this operon and experimentally confirmed the effectiveness of its influence on the expression of the *ilvBNC* operon, AHAS enzyme activity, and valine production. We demonstrated the unique functional features of this attenuator, which, due to the overlapping of the terminator and antiterminator hairpins, is capable of rapid low-energy transitions between them without the complete disruption of the hairpin structures.

## 1. Introduction

The soil bacterium *Corynebacterium glutamicum*, along with *Escherichia coli*, is one of the most commonly used basic microorganisms in creating amino acid producers, including essential branched-chain amino acids (BCAAs)—L-valine, L-leucine, and L-isoleucine. L-valine is used in food and pharmaceutical industries, medicine, and cosmetics, but primarily as a feed additive for animals. Acetohydroxy acid synthase (AHAS) is a key enzyme in the first step of the branched-chain amino acid synthesis pathway.

**Figure 1:**
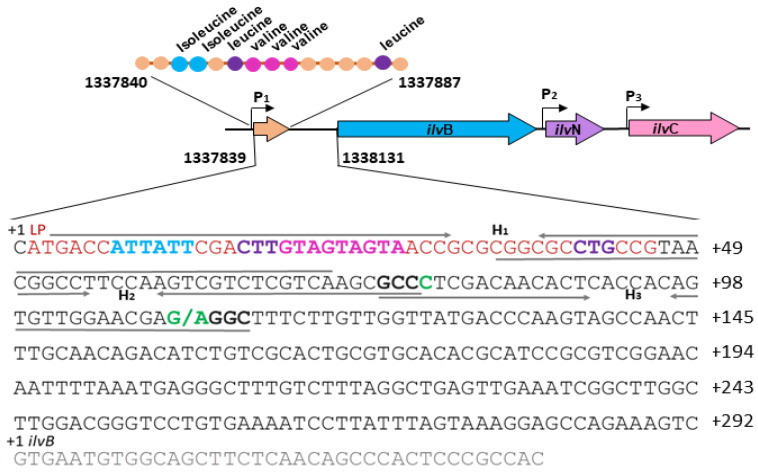
Organization of the *ilvBNC* operon and its regulatory region in *C. glutamicum* ATCC 13032. The location of the sequences of the leader peptide IlvL (marked in orange), the structural genes *ilvB*, *ilvN*, and *ilvC*, as well as the structure of the leader peptide (1337840–1337887 bp), and the 292 bp long regulatory sequence upstream of the *ilvB* gene (1337839–1338131 bp) according to sequence BA000036.3 are shown. The leader peptide sequence is marked in orange, and the regulatory codons are highlighted—isoleucine in blue, valine in crimson, and leucine in purple—and are marked in bold. Designations: P_1_, P_2_, P_3_—promoters; H_1_, H_2_, H_3_—inverted repeats indicated by intersecting arrows. The G110A mutation and its complementary nucleotide C are marked in green; the boundary trinucleotide GCC at positions 76–80 and its complementary sequence GGC at positions 111–113 are marked in bold.

In bacteria, the level of the transcription of operons providing amino acid biosynthesis is often controlled by a negative feedback mechanism through so-called attenuators [1]. The functioning of attenuators is associated with the synthesis of the leader peptide by ribosomes and the possibility of the formation of alternative secondary RNA structures in the regulatory region. When there is a lack of certain amino acids in the cell, the ribosome slows down at the codons corresponding to these amino acids, which allows the secondary structure of RNA to form antiterminator hairpins before terminator hairpins are formed. Conversely, the rapid passage of certain amino acid codons by ribosomes when there is an excess of these amino acids in the cell leads to the formation of RNA terminator hairpins that block transcription. In *C. glutamicum*, the *ilvBNC* operon, which encodes the first two enzymes of the biosynthesis of branched-chain amino acids (BCAAs: isoleucine, leucine, and valine), is subject to such regulation via a negative feedback mechanism [2]. The genes *ilvB* and *ilvN* encode subunits of the acetohydroxy acid synthase enzyme (AHAS, EC 2.2.1.6), and the *ilvC* gene encodes acetohydroxy acid isomeroreductase (AHAIR, EC 1.1.1.86) [3,4]. A low concentration of one or all three amino acids in the medium leads to a twofold increase in the activity of the operon [4]. According to current concepts, the *ilvB*, *ilvN*, and *ilvC* genes in *C. glutamicum* constitute a combined *ilvBNC* operon, which contains three active promoters (Figure 1), and their expression leads to the formation of transcripts of varying lengths—*ilvBNC*, *ilvNC*, and *ilvC* [3]. The full-length transcript *ilvBNC* starts 292 bp upstream of the first structural gene. The transcription initiation site +1 is only one nucleotide away from the start codon of the leader peptide [4]. It was also shown [4] that this 292 bp long region contains a sequence encoding the leader peptide IlvL of 15 amino acids and a region where the formation of secondary structures is possible, due to the presence of direct and reverse repeats. Morbach et al. [4] argue for the additional presence of a fourth short transcript *ilvL* approximately 200 bp in length in the region upstream of the first structural gene of the operon. The presence of a short transcript is a characteristic feature of attenuator-modulated transcriptional control. The leader peptide MTIIRLVVVTARRLP contains several codons for BCAAs: two for isoleucine (I), three for valine (V), and two for leucine (L). The replacement of three valine residues in the leader peptide with alanine residues leads to loss of valine-dependent expression, the replacement of the ATG start codon of the leader peptide with AGG reduces the expression of the operon by 80%, and a leader peptide LacZ fusion results in active beta-galactosidase [4]. All of these findings indicate the existence of a translation-dependent attenuator mechanism of transcription regulation of the *ilvBNC* operon, similar to that for the translation-dependent attenuation operons *trp* and *his* in *E. coli* and *Salmonella* [5]. The described regulatory region is a conserved sequence found in the genome of various species of the genus *Corynebacterium*, which suggests the existence of the same mechanism for regulating the expression of the *ilvBNC* operon [6]. However, the role of RNA secondary structures in the regulation of *ilvBNC* is not completely clear. It is also possible that there are additional mechanisms in the regulation of this operon, for example, a ppGpp-sensitive riboswitch mechanism [7] or an additional unknown mechanism of the transcriptional activation of *ilvBN* expression through 2-ketobutyrate [3,5].

The starting point of our study was the discovery of a mutation in the regulatory region of the *ilvBNC* operon of the *C. glutamicum* strain VF as a result of the full-genome sequencing of some *C. glutamicum* strains stored in the Russian Collection of Industrial Microorganisms (VKPM) of the National Research Center “Kurchatov Institute” (Moscow). These strains were obtained by mutagenesis and selection for their ability to produce valine. This mutation leads to the replacement of guanine with adenine at position 1337948 on the chromosome of *C. glutamicum* (GenBank: BA000036.3) [8].

The location of this mutation in the secondary structure in the regulatory region of the *ilvBNC* operon, the absence of other mutations in the structural genes of valine synthesis (*ilvB*, *ilvN*, *ilvC*, *ilvD*), and its significant effect on valine production suggest its participation in the realization of the attenuation mechanism of this operon. In this regard, it is of interest to study the structure and mechanism of operation of the attenuator of the *ilvBNC* operon in *C. glutamicum* by analyzing mutations affecting the formation of mRNA secondary structures in the attenuator region, the expression of the *ilvBNC* operon, the activity of enzymes encoded by *ilvBN* and *ilvC* genes, and valine production.

In the present work, we performed a mutational analysis of the mRNA secondary structures in the regulatory region of the *ilvBNC* operon identified previously [4,6], focusing on a region of overlapping alternative hairpin structures. The analysis showed that these hairpin structures mainly function as an antiantiterminator, antiterminator, and terminator. We proved the existence of a transcription terminator in the *ilvBNC* operon of *C. glutamicum* and experimentally confirmed its effects on the regulation of *ilvBNC* operon expression, AHAS enzyme activity, and valine production. It was also shown that the specific location of the antiterminator and terminator hairpins in the *ilvBNC* operon of *C. glutamicum* contributes to the effect of rapid switching in the attenuator, which may be important for the coordination of translation and transcription.

The reaction catalyzed by AHAS is a key point in the biosynthesis of valine and other BCAAs. The increased expression of *ilvBN* genes leads to a proportional increase in enzyme activity and, as a consequence, to an increase in valine production, and therefore is of great importance in terms of creating producer strains. Currently, the main methods for increasing *ilvBN* expression, which are used in constructing such strains, are replacing the intrinsic regulatory region of these genes with a strong constitutive promoter and introducing additional copies of the genes into cells as part of expression plasmids [2]. A deeper understanding of the intrinsic mechanisms of the regulation of the expression of these genes will allow us to find new approaches to modifying the *C. glutamicum* genome to increase BCAA production.

## 2. Materials and Methods

### 2.1. Bacterial Strains, Media, and Growth Conditions

The *E. coli* strain XL1 was grown in an LB medium at 37 °C. If necessary, antibiotics were added to the medium: kanamycin—50 µg/mL; and ampicillin—100 µg/mL. The *C. glutamicum* strains were grown in 2 × LB medium with 1% maltose at 30 °C. We used electroporation to transform DNA. A BHIS medium with the following composition was used for the growth of transformants (g/L): brain heart infusion (BHI) (Difco, Pinellas Park, FL, USA) 37.0; sorbitol—30.0; and agar—15.0. To select strains containing plasmids, kanamycin (10 μg/mL) was added to the medium. The list of *C. glutamicum* strains used in this work is presented in Table 1. The *C. glutamicum* strains ATCC 13032, VF, and L2.5 were obtained from the Russian Collection of Industrial Microorganisms (VKPM) of the National Research Center “Kurchatov Institute”, Moscow. The *C. glutamicum* strain ATCC 13869 was obtained from the German Collection of Microorganisms and Cell Cultures (DSMZ). The strains VB1, VB2, and VC2 were obtained by Derbikov D. (NCR “Kurchatov Institute”, Kurchatov Genomic Center) from *C. glutamicum* strains ATCC 13869 and ATCC 13032, by introducing a deletion into the *ponA* gene to increase the efficiency of electroporation [9] and a deletion into the *ilvA* gene (VB2, VC2) for the purpose of the further use of strains in which the synthesis of isoleucine is limited and the flow of metabolites is directed to the synthesis of valine.

To determine the level of production of BCAAs by the *C. glutamicum* strains, fermentation was carried out in test tubes in a medium with the following composition (g/L): glucose—100.0; (NH_4_)_2_SO_4_—18.9; KH_2_PO_4_—1.0; MgSO_4_ × 7H_2_O—1.0; CaCO_3_—25.0; biotin—0.0001; and thiamine—0.0002, with the addition of 50.0 mL/L of an acid hydrolyzate of wheat gluten, which was neutralized with ammonia before being added. The hydrolysis of 320 g of wheat gluten was carried out in 1 L 3.8–4.0 N sulfuric acid at 110 °C for 12 h. After hydrolysis, the precipitate was separated by filtration. The content of ammine nitrogen in the resulting hydrolyzate was not lower than 15 g/L. Test tubes with cultures were incubated for 48 h with stirring (300 rpm) at 30 °C, and then, the content of valine, leucine, and isoleucine in the culture liquid was determined by thin-layer chromatography or high-performance liquid chromatography (HPLC). The optical density at 600 nm (OD_600_) was approximately 65–72 after 48 h of fermentation for all strains.

### 2.2. The Construction of a Recombinant Integrating Plasmids and Strains of C. glutamicum with Mutations in the Regulatory Region of the ilvBNC Operon

Oligonucleotides for DNA-specific primers were synthesized by Evrogen, Moscow. The sequences of the primers are presented in Supplementary Table S1. The targeted introduction of a mutation into the regulatory region of the *ilvBNC* operon of the *C. glutamicum* chromosome was carried out by a substitution method based on homologous recombination [10]. A DNA fragment of the regulatory region of the *ilvBNC* operon, limited by primers 944-–923, containing a certain mutation, was obtained using the PCR method [11] and cloned into the plasmid pIKA-sac13, a derivative of pUC19, with the *sacB* gene from *Bacillus subtilis* 168 and the kanamycin resistance gene [12]. The plasmid pIKA-sac13 is not capable of autonomous maintenance in the host cell but can be integrated into the chromosome along homologous regions and excluded from it during growth on a medium with sucrose, leaving a mutant copy of the gene in the chromosome. The list of the plasmids carrying fragments of the regulatory region of the *ilvBNC* operon with various mutations is presented in Supplementary Table S2. *C. glutamicum* strains with mutations in the regulatory region of the *ilvBNC* operon were constructed based on *C. glutamicum* strains VB1, VB2, VC2, and L2.5 by replacing the native sequence of the regulatory region of the *ilvBNC* operon with a mutant copy.

The preparation of a competent culture of *C. glutamicum* and electroporation were carried out in accordance with the method described in [13]. The electroporation conditions were as follows: 2500 V, 2525 µF, 200 ohm. The duration of the electrical pulse was 4.5–5.5 ms, depending on the quality of the cells and the purity of the DNA. DNA sequencing was performed using an automatic ABI PRISM3500 sequencer (ThermoFisher Scientific, Waltham, MA, USA) at the Resource Centers of the NRC “Kurchatov Institute”. Km^R^ transformants were plated on a BH medium with 10% sucrose. Among the grown colonies, Km^S^ clones that lost the plasmid were selected based on the lack of growth on the BH medium with kanamycin at 10 μg/mL. Chromosomal DNA was isolated from Km^S^ colonies, and PCR was performed using primers selected to detect a particular mutation (Supplementary Table S1). “Wild” and “mutant” primers, differing in their last nucleotide, paired with primer 923, should give signals of different intensity after PCR, depending on the template sequence in the region of the replacement. If there is a mutation in the regulatory region of the *ilvB* gene, the PCR signal using the mutant primer will be more intense than that using the wild-type primer. PCR was performed with the chromosomal DNA of strain ATCC 13032 as a negative control, and with plasmid DNA used to introduce the mutation as a positive control. As a result of screening, the strains VB3, VB6, VC3, VB34, VB35, VB36, VB45, VB46, VB47, VB48, VB49, and L2.5-P*_ilvB_* (Table 1) with mutations in the regulatory region of the *ilvBNC* operon were selected. The strains VB22 and VB23 were selected among clones that did not synthesize a PCR fragment with the pair of primers 926 and 923. In all cases, the presence of mutations was confirmed by sequencing fragments amplified from the chromosomal DNA of strains using specific primers.

### 2.3. Evaluation of Gene Expression by Real-Time PCR with Reverse Transcription

The transcription level of the *ilvBNC* genes in the obtained strains was studied using real-time PCR technology. *C. glutamicum* cells were grown in BH broth until the mid-log phase. Total RNAs were isolated using the RNeasy Mini Kit (QIAGEN, Hilden, Germany). Cell lysis was performed using liquid nitrogen. To thoroughly remove genome remnants, the extracted RNA was treated with DNase I (ThermoFisher Scientific) for 30 min. cDNA synthesis was carried out using the MMLV RT kit (Evrogen, Moscow, Russia) according to the manufacturer’s protocol using a random decanucleotide primer. PCR was carried out on an ABI 7500 PCR System Fast device (Applied Biosystems, Carlsbad, CA, USA) using the qPCRmix-HS SYBR Low LowRox reagent kit (Evrogen, Moscow, Russia). PCR was carried out using primers based on the sequences of the genes being studied (Supplementary Table S1). Relative mRNA quantities were determined using the ΔΔCT method, with standard calculation algorithms provided by 7500 Fast Software version 2.3 (Applied Biosystems). As a housekeeping gene, we used the gene *fusA* coding ribosome elongation factor G. All quantifications of target genes were performed in triplicate and are presented as the mean ± standard deviation (SD).

### 2.4. Analysis of Enzymatic Activity of AHAS

The *C. glutamicum* strains were grown in flasks on a nutrient medium containing the following (g/L): KH_2_PO_4_—2.5; K_2_HPO_4_x3H_2_O—19.7; MgSO_4_ × 7H_2_O—1.0; glucose—25.0; (NH_4_)_2_SO_4_—15.0; biotin—0.0001; thiamine—0.0002; corn extract—5.0; isoleucine—0.25 (if necessary, for isoleucine auxotrophic strains); and water up to 1 L, with pH 7.0–7.2 for 17 h with stirring (300 rpm) at 30 °C, using overnight cultures of strains grown under the same conditions as the seed material. Then, the cells were collected by centrifugation, the sediment was washed with a phosphate buffer (0.1 M, pH 7.4), the cells were suspended in the same buffer with the addition of MgCl_2_ (10 mM) and glycerol (100 g/L), and they were disrupted by sonication on ice for 15 min (10 cycles—1 min of ultrasound exposure—30 s break). At the end of sonication, the samples were centrifuged at 12,000 rpm for 10 min at 4 °C. The activity of AHAS in the obtained cell-free extracts of strains was determined at 30 °C according to the described method [14,15] with some modifications. The specific activity of AHAS was calculated as nmol of α-acetolactate formed per minute per 1 mg of total protein. The amount of total protein in the extracts was determined using the Lowry method [16].

### 2.5. Bioinformatics Methods

To calculate the secondary structure of the attenuator region of the *ilvBNC* operon, the Vienna package [17] and GArna programs [18] were used. To calculate the change in the free energies of hairpins under the influence of mutations, recent thermodynamic parameters of complementary [19] and noncomplementary pairs [20] were used. To identify conserved positions in the regulatory region of the *ilvBNC* operon of *C. glutamicum*, 20 unique sequences of this region were downloaded from the NCBI server (https://blast.ncbi.nlm.nih.gov/Blast.cgi (accessed on 13 December 2024)) from the *Corynebacterium* species closest in homology to the corresponding region of the *C. glutamicum* genome. The list of species and nucleotide sequences is given in Supplementary Table S3.

## 3. Results

### 3.1. The Effect of the G110A Mutation in the Regulatory Region of the ilvBNC Operon in C. glutamicum Strains

The initial premise of this research was the detection of a mutation in the *C. glutamicum* VF strain with increased valine production—a substitution of guanine with adenine at a position −183 bp upstream of the start of translation of the *ilvB* gene. As shown in Figure 1, the leader peptide is located upstream of the structure gene of the *ilvBNC* operon at position 1337840–1337887 bp according to sequence BA000036.3. The point mutation discovered during the whole-genome sequencing of the valine-producing strain *C. glutamicum* VF (replacement of G with A) is located within the regulatory region at position 1337948 (GenBank: BA000036.3)—183 bp upstream of the start codon of the *ilvB* structural gene outside the leader peptide sequence [8], or 110 bp downstream of the transcription start codon. The *C. glutamicum* VF strain is derived from the *C. glutamicum* ATCC 13032 strain and does not have other mutations in the structural genes for valine synthesis (*ilvB*, *ilvN*, *ilvC*, *ilvD*) [8].

The G110A mutation was introduced into various strains originating from *C. glutamicum* ATCC 13032 and *C. glutamicum* ATCC 13869, as well as into the *C. glutamicum* L2.5 strain, a leucine producer obtained by mutagenesis, presumably from the *C. glutamicum* ATCC 14067 strain. From the results presented in Table 2, it follows that the G110A mutation in all studied strains of *C. glutamicum* leads to an increase in valine production from 50% to several times compared to the production of the original strain. The G110A mutation did not affect the growth rate of the strains. The optical density at 600 nm (OD_600_) for all strains was approximately 65–72 after 48 h of fermentation. It is interesting to note that the potential of the *C. glutamicum* strains ATCC 13032 and ATCC 13869 also differs in the level of valine production: a derivative of *C. glutamicum* ATCC 13869 produces five times more valine than a derivative of *C. glutamicum* ATCC 13032.

Enzymes encoded by the *ilvB*, *ilvN*, and *ilvC* genes are involved in the biosynthesis of all three BCAAs: valine, leucine, and isoleucine. One would expect that the G110A mutation in the regulatory region of the *ilvBNC* operon would affect the production of not only valine but also other BCAAs. However, as shown in Table 2, the presence of the G110A mutation in the studied *C. glutamicum* strains had virtually no effect on the production of isoleucine and had no significant effect on the production of leucine, even in a strain whose metabolism is aimed at its synthesis. The latter may be related to the possibility that the rate-limiting enzyme is not AHAS but another enzyme in the leucine biosynthesis chain.

**Figure 2:**
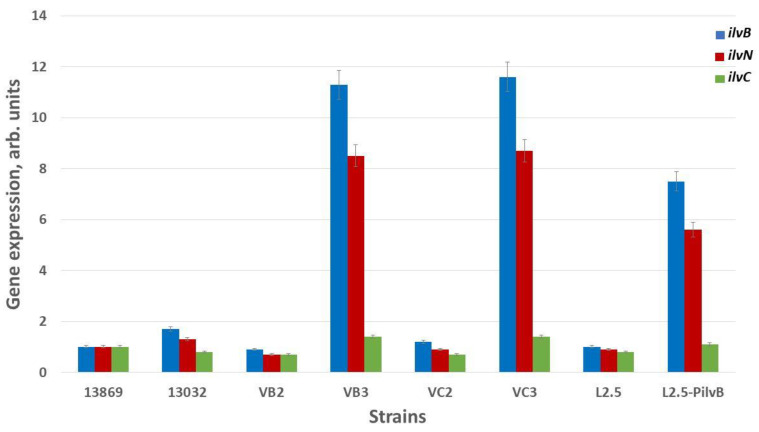
Effect of the G110A mutation on the transcription level of the *ilvB*, *ilvN*, and *ilvC* genes in different strains of *C. glutamicum*. The level of gene expression in the strain ATCC 13869 is set to 1. Data are calculated as the average of three experiments. Error bars show standard deviations.

The level of transcription of the *ilvB*, *ilvN*, and *ilvC* genes in the strains with mutations in the regulatory region of the *ilvBNC* operon was studied using real-time RT-PCR technology. From the results presented in Figure 2, it can be seen that the introduction of the G110A mutation into the genome of the different strains of *C. glutamicum* leads to a several-fold increase in the transcription of the *ilvB* and *ilvN* genes encoding the subunits of the AHAS enzyme, which in turn leads to an increase in AHAS activity by 6.5 times compared to the parental strains (Table 2). These facts suggest the involvement of the mutation G110A in the attenuator regulation of the transcription of the *ilvBNC* operon.

### 3.2. A Model for the Transcriptional Regulation of the C. glutamicum ilvBNC Operon and the Computer Modeling of Mutations in It

We noted above that both Morbach et al. [4] and Narunsky et al. [6] suggested the possibility of the formation of three hairpins in the regulatory region of the *ilvBNC* operon in *C. glutamicum*, one of which was positioned as a terminator, and the possibility of its formation was confirmed by enzymatic probing [6], but its functionality as a transcription terminator was not proved experimentally, because the deletion of a part of this hairpin did not significantly affect the expression of the operon [4]. The role of the other two hairpins in the mechanism of regulation of the expression of the *ilvBNC* operon also remains incompletely understood.

**Figure 3:**
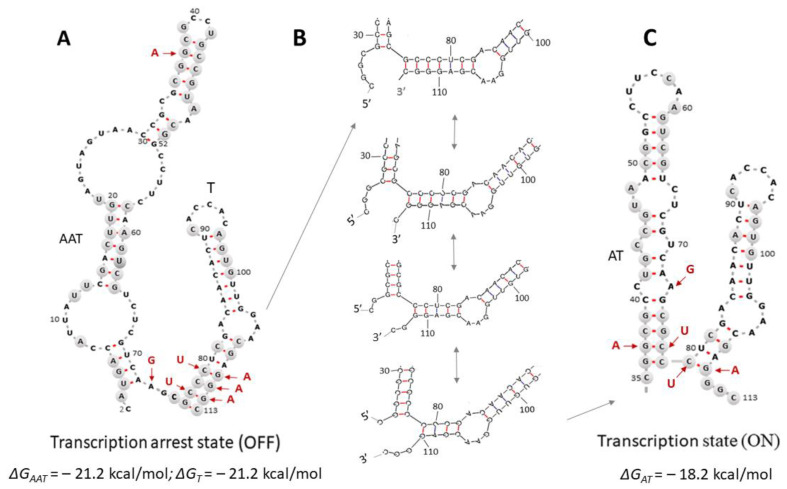
Thermodynamically stable mRNA secondary structures, the formation of which is possible in the regulatory region of the *ilvBNC* operon in *C. glutamicum*. (**A**)—state of stopped transcription; (**B**)—intermediate structures responsible for the rapid transition between the terminator and antiterminator hairpins by the low-energy switching of nucleotides 76–78 from pairing with nucleotides 111–113 to pairing with nucleotides 36–38; (**C**)—state of transcription. Red arrows indicate the positions of single-nucleotide substitutions. Positions conserved in the attenuator sequences of the 19 *Corynebacterium* species closest to *C. glutamicum* are marked in gray. Denotations: T—terminator hairpin; AT—antiterminator hairpin; AAT—antiantiterminator hairpin.

To analyze the mechanism of transcription attenuation in the regulatory region of the *ilvBNC* operon, we performed computer calculations of the secondary structure of the attenuator using the programs described in [17,18]. The calculations confirmed the possibility of formation in the regulatory region of the *ilvBNC* operon from the three inverted repeats H_1_, H_2_, and H_3_ (Figure 1) of the two most thermodynamically stable mRNA secondary structures, which correspond to the two regulatory states (Figure 3A,B). In the first case, the hairpin H_1_ blocks the formation of hairpin H_2_ and does not prevent the formation of the terminator hairpin H_3_, which leads to transcriptional arrest, and the OFF state occurs (Figure 3A). In the second case, H_2_ hairpin formation prevents the formation of the terminator hairpin H_3_, as their sequences overlap with each other, and the ON transcription state occurs (Figure 3B). Thus, in terms of role assignment, the H_1_ hairpin is an antiantiterminator and the H_2_ hairpin is an antiterminator. Antiantiterminator structures in bacterial attenuators have been found previously [21].

It should be noted that the two states described above are difficult to distinguish in comparative analysis and enzymatic probing [6] because the paired nucleotides in the ON and OFF states are often the same.

It was noted above that the start of translation in the *ilvBNC* operon is only one nucleotide away from the start codon of the leader peptide [4]. For bacterial translation initiation, such peptides are characterized by the landing of the ready ribosomal complex in the start codon region, which requires 10–11 nucleotides downstream from the start codon free of complementary interactions [22]. This feature casts doubt on whether the H_1_ hairpin can exist in a state of initiated translation, because the H_1_ hairpin shields the ribosome landing region and thus physically blocks translation initiation. However, given that the H_1_ hairpin is located in a region that is conserved in the *ilvBNC* operon in *Corynebacterium* [6], the role of this hairpin remains unclear and may not be related to an attenuation mechanism as in some bacterial species in which the expression of the *ilvBNC* operon is controlled by a ppGpp-sensitive riboswitch mechanism [7].

Given the above-mentioned data and the fact that the enzymatic probing of the secondary structure of the region of interest in *C. glutamicum* was performed on another *Corynebacterium* species, *C. ulcerans*, we compared the sequences in a narrower sample of *Corynebacterium* species than that in [6], namely, in the 20 species in which the sequences of this region are closest to those of *C. glutamicum*, including *C. glutamicum*. Thus, the nucleotide positions in the regulatory region of the *ilvBNC* operon that are conserved in the sample of *Corynebacterium* species closest to *C. glutamicum* were identified. However, as can be seen in Figure 3, where these positions are marked in gray, the conserved nucleotides at positions 36-64 are equally involved in both H_1_ (AAT) and H_2_ (AT) hairpin formation, making it impossible to confidently distinguish between them using evolutionary and enzymatic analyses.

All three hairpins, AAT, AT, and T, have comparable stability (Figure 3), but the AT and AAT hairpins unfold as a result of interaction with the ribosome. Therefore, to a first approximation, changes in the gene transcription level caused by mutations in the attenuator will be proportional to the mutational perturbations of terminator stability. More specifically, to describe the mechanism of attenuation by a structural model (Figure 3), we compared the change in the transcription level of the *ilvBNC* operon with the change in H_3_ hairpin free energy as influenced by base pairing changes. The difference between the hairpin free energies was calculated on the basis of the thermodynamic parameters of the energies of noncomplementary pairs [19,20] using the formula

ΔΔG = ΔG_mT_ − ΔG_T_ (1)

where ∆G_mT_ and ∆G_T_ are the free energies of the mutated and the original hairpins, T, shown in Figure 3A.

To analyze terminator functionality, mutations were introduced into the attenuator structure, which mainly affected conserved positions in the overlap region of the antiterminator and terminator hairpins, H_2_ and H_3_. The list of mutant strains and the positions of the replaced nucleotides are summarized in Table 1 (see also Figure 3).

Calculations of the change in the energies of terminator hairpin formation in the attenuator of the *ilvBNC* operon under the influence of mutations and experimental data on the effect of the latter on the expression of the *ilvBNC* operon, AHAS activity, and valine production are given in the next section.

### 3.3. Experimental Confirmation of a Computer Model of the Transcriptional Regulation of the C. glutamicum ilvBNC Operon

We chose a strategy using single substitutions that form or eliminate single noncomplementary pairs in hairpins. Figure 3 shows that the conserved pair of tetranucleotides GCCC (positions 76–79) and GGGC (positions 110–113) are involved in terminator hairpin formation, and the boundary GCC trinucleotide (positions 76–78) is also involved in antiterminator hairpin formation by forming complementary bonds with the GGC trinucleotide (positions 36–38). On this basis, strains were created with 11 point mutations in the seven positions. We introduced mutations of three types: (1) at positions 110–112, which are responsible for the stability of only the terminator hairpin in the critical region, (2) at positions 77 and 79, which are involved in complementary interactions of the critical region of both the terminator and antiterminator, and (3) at positions 37 and 73, which are involved in the antiterminator and antiantiterminator but not the terminator. We also made two double mutations by combining mutations from different groups.

According to the calculated values of changes in terminator hairpin stability (Table 3), we can expect that the level of valine production will decrease with increasing group number, with double mutations falling into the second group. Deviations from the general trend would characterize the contribution of short-living antianti- and antiterminator secondary structures.

**Figure 4:**
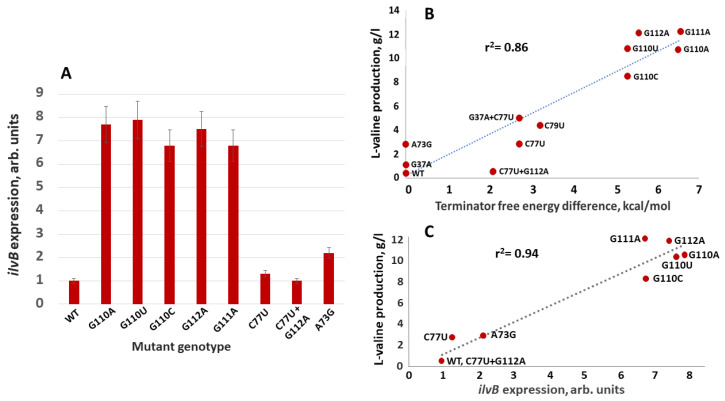
Correlation between *ilvB* gene expression levels, production of valine, and energy change in terminator hairpin in mutant strains. (**A**) The relative level of expression of the *ilvB* gene in the *C. glutamicum* strains with mutations in the terminator–antiterminator region. The level of gene expression in the strain VB1 (wt) is set to 1. Strains with the mutations G37A, G37A+C77U, and C79U were not tested in this experiment. (**B**) The dependence of the level of valine production on the changes in free energy of the T hairpin. (**C**) The dependence of the level of valine production on the relative level of expression of the *ilvB* gene in the mutant strains. Data of *ilvB* expression and valine production are calculated as the average of three experiments. Error bars show standard deviations.

Overall, the experimental data support the hypothesis and are in reasonably good agreement with each other; the high correlation coefficient (r^2^ = 0.86, *p* < 0.05, Figure 4B) indicates that variations in valine production in mutant strains are largely due to changes in terminator stability alone.

The first group of mutations, G110C, G110T, G110A, G111A, and G112A, weakens the terminator structure by disrupting complementarity with the CCC trinucleotide (positions 77–79) at the base of the hairpin, and increases valine synthesis by an order of magnitude (Table 3).

In the second group, C77T, C79T, G37A+C77T, and C77T+G112A, the picture is more complex. Three of them, G37A+C77T, C77T, and C79T, slightly destabilize the terminator by replacing the strong G-C pair with the weaker A-U and G-U pairs, resulting in a level of valine production near the regression line (Figure 4B). The combination of C77T and G112A mutations restore the expression of the *ilvBNC* operon as in the wild type.

The A73G and G37A mutations from the third group do not affect the terminator and thus have a moderate-to-weak effect on valine synthesis (Table 3, Figure 4B). From Figure 4B, we can estimate the effective stabilization of the antiterminator as a result of the A73G mutation as −1.5 kcal/mol in free energy, which is much weaker than the value of −6.8 kcal/mol calculated for the hairpin antiterminator from the thermodynamic parameter tables.

Experimental data for measuring the level of expression of the *ilvB* gene in *C. glutamicum* strains with simulated mutations in the terminator–antiterminator region are presented in Figure 4. It should be noted that all mutations in the regulatory region did not affect the growth rate of the strains.

We observed a high correlation (r^2^ = 0.94, *p* < 10^−3^) between the level of valine production and the level of *ilvB* gene expression (Figure 4C), indicating a key contribution of *ilvB* gene transcription to the control of valine production in *C. glutamicum* under these cultivation conditions. The levels of *ilvB* gene expression and valine production also correlated well with the level of AHAS enzyme activity, with r^2^ = 0.89 and r^2^ = 0.92, respectively, as indicated by a comparison of the data in Figure 4A and in Table 3.

A significant correlation (r^2^ = 0.86, *p* < 0.05, Figure 4B) was also observed between the level of valine production of the *ilvBNC* operon in *C. glutamicum* mutant strains and the difference in terminator free energy ΔΔG according to the structural model presented in Figure 3.

Taken together, our results support our proposed model for the regulation of the expression of the *ilvBNC* operon in *C. glutamicum*, based on a low-energy switch between terminator and antiterminator hairpins, due to the fact that the boundary trinucleotide GCC can participate in the formation of both hairpins.

## 4. Discussion

**Figure 5:**
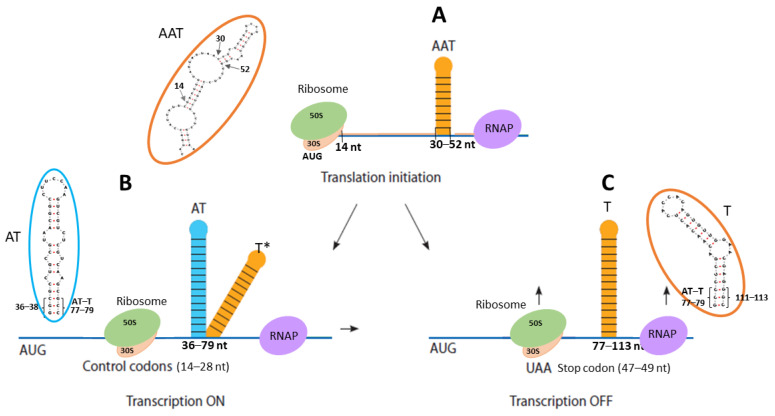
Schematic of attenuator functioning in the *ilvBNC* operon of *C. glutamicum*. (**A**)—transcriptional pause state, when the upper part of the AAT hairpin (positions 30–52) interacts with RNA polymerase and creates conditions for ribosome landing and translation initiation; (**B**)—state of translational pause, when the ribosome delays the control codons and creates conditions for the formation of the AT hairpin (positions 36–79) and transcription; (**C**)—state of transcriptional termination, when the ribosome quickly passes the control codons, and instead of AT, the T hairpin is formed (positions 77–113), which completes transcription. Denotations: AAT—antiantiterminator; AT—antiterminator; T—terminator; T*—unstable terminator when its GCC boundary trinucleotide has bound to form the AT structure. Brackets indicate the position of the GCC boundary trinucleotide at positions 77–79, which is involved in the AT and T structures. The AAT and T hairpins that block translation and transcription are marked in orange, and the AT hairpin that allows them is marked in blue.

Due to its small size, the regulatory region of the *ilvBNC* operon in *C. glutamicum* is saturated with functional elements controlling translation, transcription, and their coordination. Our study focused on the role of RNA secondary structures in this region. Three hairpins can be formed in it, two of which, H_2_ and H_3_ (Figure 1), correspond to the antitermination (Figure 3C) and termination states of transcription (Figure 3A) according to [4]. The H_1_ hairpin (Figure 1) stops both translation and transcription because it (a) blocks translation initiation and (b) does not prevent the formation of the terminator H_3_ hairpin; i.e., it is essentially an antiantiterminator. Situation (a) is related to the peculiarities of the translation initiation of leaderless bacterial mRNAs, which begins with the recognition of the start codon by the assembled ribosome [22]. An mRNA is considered available for translation initiation if it is free of complementary interactions throughout the length of its ribosome coverage downstream of the start codon [22]. The total length of ribosome coverage in bacteria was found to be twenty-four nucleotides (with a movement step of one nucleotide, as opposed to one codon in eukaryotes). That is, in the *ilvBNC* operon of *C. glutamicum*, the formed H_1_ hairpin shields the translation initiation site. We assume that the necessary condition for the translation of the leader peptide is the synchronization of translation and transcription rates in the leader region of the operon; namely, the formation of the H_1_ hairpin starts from its apex (positions 30–52) as the nucleotide sequence is synthesized by the polymerase. The apex of the H_1_ hairpin interacts with the polymerase and creates a transcriptional pause during which the initiation site region (positions 2–29) remains open for translation initiation by the ribosome (Figure 5A). The ribosome then reaches the control codons CUU (leucine codon) and GUA (valine codon), the importance of which is emphasized by their conservativity among the closest *C. glutamicum* homologs. If it passes them rapidly, the H_2_ antiterminator hairpin does not have time to assemble and the H_3_ terminator hairpin is formed, which terminates transcription (Figure 5C). When paused at the control codons, the antiterminator hairpin H_2_ is formed instead of the terminator hairpin H_3_, which activates transcription by rapidly displacing complementary interactions in the terminator (Figure 5B). Rapid switching is critical because of the short distance between the control codons and positions 36–38, whose nucleotides displace nucleotides at positions 111–113 of the terminator during antiterminator formation (Figure 3B). In other words, the binding of the boundary trinucleotide GCC is critical for the selection of an antitermination or termination state because this trinucleotide can be partially incorporated into both hairpins, and the minima of antitermination and termination states on the conformational landscape are connected by a low-energy valley that can be rapidly traversed without completely disrupting one of the hairpins. As a result, the regulation of transcription in the operon under consideration should be hypersensitive and fast, and transcriptional noise caused by fluctuations in secondary structures should be suppressed. Such an attenuator can be called a “fast” attenuator, the functional role and evolutionary origin of which require a separate study.

The mutation at position 110 found in the *C. glutamicum* VF strain obtained by random mutagenesis and selection for valine formation is located at the base of the terminator hairpin within the GGGC tetranucleotide (positions 110–113) complementary to the boundary trinucleotide GCC and is not involved in the formation of the antiterminator hairpin (Figure 3).

Since terminal pairs of hairpin nucleotides are usually prone to the decay of complementary bonds, this model suggests that the presence of mutation 110 disrupts the secondary structure of the terminator and contributes to the fact that the boundary GCC trinucleotide escapes into the hairpin structure of the antiterminator, forming complementary bonds with the GGC trinucleotide (positions 36–38). Moreover, this mutation also creates a single-nucleotide spacer between the hairpins in the antitermination state and thus partially blocks the continuous transition to the terminator state.

Thus, we believe that although the conserved regulatory region of the *ilvBNC* operon in *Corynebacterium* identified by Narunsky et al. [6] is capable of forming mRNA structures that play the role of a terminator, antiterminator, and antiantiterminator, the role of the latter in transcriptional attenuation is related to two aspects: (a) the transcription pausing that triggers translation and creates conditions for attenuation through the competition between antiterminator and terminator structures, and (b) the conservativity of the leader peptide containing codons of controlling amino acids on which ribosomes pause in case of their deficiency. Contrary to the comparable stability of AAT, AT, and T hairpins, the active interaction of the AAT and AT regions with the ribosome causes the stability of the AAT and AT hairpins to be inferior to that of the T hairpin in its contribution to operon expression.

In conclusion, it should be added that Morbach et al. [4] hypothesized essentially the same sequences for hairpins as we did, but they did not prove the functionality of the terminator structure or discuss the importance of the overlap of these two sequences for the mechanism of regulation. The mutations obtained by Morbach et al. [4], named #11, #15, and #20, were located outside the T-AT region we examined and showed approximately the same gene expression as the wild-type strain, and therefore cannot provide evidence for terminator functionality. The mutations that we introduced into strains VB6, VB22, VB23, VB35, and VB46 are located in the T-AT region and are involved in terminator stem formation. As we have shown, these mutations lead to a seven- to eight-fold increase in the expression level of the operon compared with the wild-type strain, which, on the one hand, indicates the importance of the T-AT structure in attenuator function and, on the other hand, proves the functionality of the terminator.

The fact that the regulatory region of the *ilvBNC* operon retains its structure in 103 species belonging to the genus *Corynebacterium* [6], with the terminator sequence being the most conserved part of it, suggests the possibility of the existence of the “fast” attenuation mechanism we proposed for the *ilvBNC* operon of *C. glutamicum* in all species examined by Narunsky et al. [6].

Conformational changes in attenuator mRNA are dynamic, associated with translation, and depend on the influence of many other factors, the establishment of which is a topic for further research. The role of the leader peptide in the regulation of operon expression is not completely clear, and the same can be said about the effect of 2-ketobutyrate on enhancing expression. The model we propose does not take these factors into account; however, it nevertheless has high predictive value and can be used both in studying the regulation of the expression of the *ilvBNC* operon and in the targeted modification of the genome to increase valine production in *C. glutamicum* strains.

## 5. Conclusions

The mutational analysis of mRNA secondary structures in the regulatory region of the *ilvBNC* operon of *C. glutamicum* allows us to propose a model of the regulation of its transcription with the participation of three mRNA secondary structures, which essentially play the roles of an antiantiterminator, antiterminator, and terminator, and demonstrates the effectiveness of the influence of the terminator structure on the regulation of *ilvBNC* operon expression, AHAS enzyme activity, and valine production.

Computer calculations of the formation of secondary structures in the attenuator region of the *C. glutamicum ilvBNC* operon have shown that the bases of the two thermodynamically stable structures—the transcription terminator hairpin and the antiterminator hairpin—are adjacent to each other and overlap at a trinucleotide GCC. The choice between the termination and antitermination of transcription comes down to competition between hairpin bases for binding to the boundary trinucleotide GCC at positions 76–78, which is complementary to the GGC trinucleotides at positions 111–113 and GGC at positions 36–38 (Figure 1). The presence of a boundary trinucleotide, which is important for the formation of both hairpins, is a hallmark of the *ilvBNC* operon. Because of this feature, the attenuator becomes capable of rapid, low-energy transitions from one state to another without the complete disruption of base pairing. This is a new type of attenuator that can be called “fast”. Our model is supported by experimental data and has high predictive value. All mutations located at the base of the terminator hairpin GGGC (positions 110–113) disrupt complementarity with the tetranucleotide GCCC (positions 76–79) and lead to the activation of the expression of *ilvBN* genes, an increase in the activity of the key enzyme of valine synthesis—AHAS—and an increase in the production of valine. These results expand our knowledge of the transcriptional regulation of the *ilvBNC* operon and may facilitate the development of valine-producing strains.

## 6. Patents

Patent RU 2753996 C1. 2021. Ryabchenko, L.E.; Gerasimova, T.V.; Leonova, T.E.; Kalinina, T.I.; Sheremetieva, M.E.; Anufriev, K.E.; Yanenko A.S. Bacterium *Corynebacterium glutamicum* with increased ability to produce L-valine and method for producing L-valine using this bacterium.

## Figures and Tables

**Table 1:** The strains of *C. glutamicum* used in this work.

Strain	Strain Characteristics	Source
DSM 1412 (ATCC13869)	Wild-type strain	Collection of microorganisms DSMZ
ATCC13032	Wild-type strain	VKPM
VF	Valine producer (mutagenesis)	VKPM, B5212
VB1	ATCC 13869 Δ*ponA*	Received by Derbikov D.
VC2	ATCC 13032 Δ*ponA* Δ*ilvA*	Rceived by Derbikov D.
VC3	VC2 G110A	Received by Derbikov D.
VB2	VB1 Δ*ilvA*	Received by Derbikov D.
VB3	VB2 G110A	This work
VB6	VB1 G110A	This work
VB22	VB1 G110T	This work
VB23	VB1 G110C	This work
VB34	VB1 A73G	This work
VB35	VB1 G111A	This work
VB36	VB1 C77T	This work
VB45	VB1 C77T; G112A	This work
VB46	VB1 G112A	This work
VB47	VB1 G37A	This work
VB48	VB1 G37A; C77T	This work
VB49	VB1 C79T	This work
L2.5	Leucine producer (mutagenesis)	VKPM
L2.5-P*_ilvB_*	L2.5 G110A	This work

**Table 2:** Effect of the G110A mutation in the regulatory region of the *ilvBNC* operon at the level of BCAA biosynthesis and AHAS activity in different strains of *C. glutamicum.*

Strain	Valine ^1^, g/L	Leucine ^1^, g/L	Isoleucine ^1^, g/L	AHAS Activity ^2^, Units/mg Protein
VC2	3.6 ± 0.2	0	0	1.6 ± 0.3
VC3	15.6 ± 0.5	0	0	10.5 ± 1.5
VB1	0.44 ± 0.10	0	0	13.5 ± 1.5
VB2	16.2 ± 0.5	0.5 ± 0.1	0	21.5 ± 2.5
VB3	24.1 ± 1.0	1.0 ± 0.1	0	140.0 ± 15.0
L2.5	0	10.0 ± 0.3	0.5 ± 0.1	ND ^3^
L2.5-P*_ilvB_*	15.1 ± 0.5	10.0 ± 0.3	0.5 ± 0.1	ND ^3^

^1^ The levels of production of BCAAs are calculated as the average of three experiments. ^2^ Activity values are calculated as the average of three experiments. ^3^ ND—no data.

**Table 3:** Effects of mutations in the regulatory region of the *ilvBNC* operon on the properties of *Corynebacterium* strains.

Mutant Genotype	Valine ^1^ (g/L)	AHAS Activity, Units/mg Protein ^2^	Terminator Free Energy Difference, kcal/mol
ATCC13869 Δ*ponA*	0.4 ± 0.2	13.0 ± 1.5	0
G111A	12.2 ± 0.5	116.2 ± 10.0	6.56
G112A	12.1 ± 0.5	86.9 ± 8.5	5.56
G110A	10.7 ± 0.5	84.5 ± 8.5	6.49
G110T	10.8 ± 0.5	91.5 ± 9.5	5.29
G110C	8.5 ± 0.5	83.5 ± 8.5	5.29
A73G	2.8 ± 0.2	40.1 ± 4.0	0
C77T	2.8 ± 0.2	23.4 ± 2.0	2.71
C77T+G112A	0.5 ± 0.2	19.1 ± 2.0	2.07
G37A	1.1 ± 0.2	20.0 ± 2.0	0
G37A+C77T	5.0 ± 0.25	52.0 ± 4.0	2.71
C79T	4.4 ± 0.25	22.0 ± 2.0	3.20

^1^ The levels of production of valine are calculated as the average of three experiments. ^2^ Activity values are calculated as the average of three experiments.

## Data Availability

Date are contained within the article and supplementary materials.

## Supplementary Materials

The following supporting information can be downloaded at https://www.mdpi.com/article/10.3390/microorganisms13020291/s1, Table S1. DNA-specific primers used in this work. Table S2. Recombinant plasmids based on the pIKA-sac13 vector with the mutant regulatory region of the *ilvBNC* operon of *C. glutamicum*. Table S3. The alignments of the leader region of the *Corynebacterium ilvBNC* operon.
