# Supplementary material for: Mutational Analysis Supports Three-Hairpin Model of Attenuator for Transcription Regulation of ilvBNC Operon in Corynebacterium glutamicum
Source: Microorganisms. 2025 Jan 28;13(2):291. doi: 10.3390/microorganisms13020291 (PMC11857589; doi:10.3390/microorganisms13020291)
Supplement: Supplementary file 1 [file microorganisms-13-00291-s001.zip › microorganisms-3399925-supplementary.pdf]

Supplementary matherial

Mutational analysis supports three-hairpin model of attenuator for transcription regulation of *ilvBN*C operon in *Corynebacterium glutamicum*

Ludmila E. Ryabchenko[1]*, Igor I. Titov[2,3], Tatyana E. Leonova[1], Tatyana I. Kalinina[1], Tatyana V. Gerasimova[1], , Marina E. Sheremetieva[1], Nikolay A. Kolchanov[2,3], Tamara M. Khlebodarova [2,3], Alexander S. Yanenko[1]

Author affiliations:
[1] National Research Center "Kurchatov Institute", Kurchatov Genomic Center, 123182, Akademika Kurchatova pl. 1, Moscow, Russia
[2] Department of Systems Biology, Institute of Cytology and Genetics SB RAS, 630090, Akademika Lavrentyev Ave., 10, Novosibirsk, Russia
[3] Kurchatov Genomic Center, Institute of Cytology and Genetics, SB RAS, 630090, Akademika Lavrentyev Ave., 10, Novosibirsk, Russia

*Corresponding author: Ludmila E. Ryabchenko[1], e-mail l.ryabchenko@mail.ru

Table S1. DNA-specific primers used in this work.

Primer	Sequence	Purpose
944	aagtccgcaa caatcgactgcg	To amplify the *ilvBNC* regulatory region
923	tttgaccatgaagttgtgctt	
926	cacagtgttggaacgag	To identify the mutations
927	cacagtgttggaacgaa	G110T, G110T, G110C
1033	ccacagtgttggaacgatggctttcttgttggttatg	To obtain a construct with the mutation
1034	cataaccaacaagaaagccatcgttccaacactgtgg	G110T
1031	ccacagtgttggaacgacggctttcttgttggttatg	To obtain a construct with the mutation
1032	cataaccaacaagaaagccgtcgttccaacactgtgg	G110C
1045	ccaagtcgtctcgtcaggcgccctcgacaacact	To obtain a construct with the mutation
1046	agtgttgtcgagggcgcctgacgagacgacttgg	A73G
1047	aagtcgtctcgtcaagcgtcctcgacaacactca	To obtain a construct with the mutation C77T
1048	ccaca tgtggtgagtgttgtcgaggacgcttgacgagacgactt	
1049	accacagtgttggaacgagagctttcttgttggttatg	To obtain a construct with the mutation
1050	cataaccaacaagaaagctctcgttccaacactgtggt	G111A
1057	ccaagtcgtctcgtcag	To identify the mutation A73G
1058	ccaagtcgtctcgtcaa	
1059	aagtcgtctcgtcaagcgt	To identify the mutation C73T
1060	aagtcgtctcgtcaagcgc	
1061	cacagtgttggaacgaga	To identify the mutation G111A
1062	cacagtgttggaacgagg	
1073	accacagtgttggaacgaggactttcttgttggttatg	To obtain a construct with the mutation
1074	cataaccaacaagaaagtcctcgttccaacactgtggt	G112A
1075	agtgttggaacgagga	To identify the mutation G112A
1076	agtgttggaacgaggg	

1169	gtagtaaccgcgcgacgcctgccgtaacgg	To obtain a construct with the mutation G37A
1170	ccgttacggcaggcgtcgcgcggttactac	
1171	ttgtagtagtaaccgcgcaa	To identify the mutation G37A
1172	ttgtagtagtaaccg cgcgg	
1173	gtcgtctcgtcaagcgccttcgacaacactcaccaca	To obtain a construct with the mutation C79T
1174	tgtggtgagtgttgtcgaaggcgcttgacgagacgac	
1175	gtcgtctcgtcaagcgccc	To identify the mutation C79T
1176	gtcgtctcgtcaagcgct	
fusA F	aatcggtgctgaggacaacttc	For endogenous control in real-time PCR
fusA R	ggtaactccacgccaggtca	
ilvB F	ccagtgaccaagcacaactt	For the *ilvB* transcript real-time PCR
ilvB R	attactggtcgccctggtcc	
ilvN F	acctcgtgtccctcgtgtct	For the *ilvN* transcript real-time PCR
ilvN R	tgcgacttgatgaagagacc	
ilvC F	tgatgctgacgctgacctct	For the *ilvC* transcript real-time PCR
ilvC R	attctggcgttgaggttgtc	

Table S2 Recombinant plasmids based on the pIKA-sac13 vector with the mutant regulatory region of the *ilvBNC* operon of *C. glutamicum*.

Plasmids	Primers	The strains that are the sources of template DNA
pIKA P_{ilvB}^{G76A}	944 + 923	VF
pIKA P_{ilvB}^{G76T}	(944+1034) and (1033+923)	ATCC13032
pIKA P_{ilvB}^{G76C}	(944+1032) and (1031+923)	ATCC13032
pIKA P_{ilvB}^{G77A}	(944+1050) and (1049+923)	ATCC13032
pIKA P_{ilvB}^{G78A}	(944+1074) and (1073+923)	ATCC13032
pIKA P_{ilvB}^{A39G}	(944+1046) and (1045+923)	ATCC13032
pIKA P_{ilvB}^{C43T}	(944+1048) and (1047+923)	ATCC13032
pIKA $P_{ilvB}^{\Delta 57-79}$	(944 +942) and (943+ 923)	VF
pIKA P_{ilvB}^{G3A}	(944+1170) and (1169+923)	ATCC13032
pIKA $P_{ilvB}^{G3A; C43T}$	944+1170) and (1169+923)	VB36

Table S3. The alignments of the leader region of the *Corynebacterium ilvBNC* operon

Description	Acc. Len	Accession
Corynebacterium glutamicum acetohydroxy acid synthase (ilvB) a...	4705	L09232.1
Corynebacterium glutamicum DNA, complete genome, strain: N24	3537507	AP017369.1
Corynebacterium deserti GIMN1.010, complete genome	2972149	CP009220.1
Corynebacterium crudilactis strain JZ16 chromosome, complete...	3047373	CP015622.1
Corynebacterium callunae DSM 20147, complete genome	2839551	CP004354.1
Corynebacterium faecale strain DSM 45971 chromosome, complete...	2987260	CP047204.1
Corynebacterium occultum strain 2039 chromosome, complete genome	3109186	CP046455.1
Corynebacterium vitaeruminis DSM 20294, complete genome	2931780	CP004353.1
Corynebacterium hindlerae strain NML 93-0612 chromosome	2680694	CP059833.1
Corynebacterium sp. SCR221107 chromosome, complete genome	3043024	CP115670.1
Corynebacterium humireducens NBRC 106098 = DSM 45392, complete...	2681312	CP005286.1
Corynebacterium halotolerans YIM 70093 = DSM 44683, complete...	3135752	CP003697.1
Corynebacterium ulcerans strain MRi49 chromosome, complete genome	2527244	CP046863.1
Corynebacterium silvaticum strain PO100/5 chromosome, complete...	2572864	CP021417.2
Corynebacterium durum strain DSM 45333 chromosome, complete...	2786863	CP047200.1
Corynebacterium pseudotuberculosis strain PA09 chromosome	2338000	CP054555.1
Corynebacterium poyangense strain 4H37-19 chromosome, complete...	2617997	CP046884.1
Corynebacterium diphtheriae strain CN2000 chromosome	2530683	CP039522.1
Corynebacterium gerontici strain W8 chromosome, complete genome	2263210	CP033897.1
Corynebacterium frankenforstense DSM 45800 strain ST18, comple...	2604152	CP009247.1

Alignments:
>Corynebacterium glutamicum acetohydroxy acid synthase (ilvB) and (ilvN) genes, and acetohydroxy acid isomeroreductase (ilvC) gene, complete cds
Sequence ID: L09232.1 Length: 4705
Range 1: 459 to 571
Score:205 bits(226), Expect:2e-51,
Identities:113/113(100%), Gaps:0/113(0%), Strand: Plus/Plus
Query 1
ATGACCATTATTCGACTTGTAGTAGTAACCGCGCGGCGCCTGCCGTAACGGCCTTCCAAG 60
||||||||||||||||||||||||||||||||||||||||||||||||||||||||||||
Sbjct 459
ATGACCATTATTCGACTTGTAGTAGTAACCGCGCGGCGCCTGCCGTAACGGCCTTCCAAG
518
Query 61
TCGTCTCGTCAAGCGCCCTCGACAACACTCACCACAGTGTTGGAACGAGGGCT 113
|||||||||||||||||||||||||||||||||||||||||||||||||||||
Sbjct 519
TCGTCTCGTCAAGCGCCCTCGACAACACTCACCACAGTGTTGGAACGAGGGCT 571

>Corynebacterium glutamicum DNA, complete genome, strain: N24
Sequence ID: AP017369.1 Length: 3537507
Range 1: 1491940 to 1492052
Score:196 bits(216), Expect:1e-48,
Identities:111/113(98%), Gaps:0/113(0%), Strand: Plus/Plus
Query 1
ATGACCATTATTCGACTTGTAGTAGTAACCGCGCGGCGCCTGCCGTAACGGCCTTCCAAG 60
||||||||| ||||||||||||||||||||||||||||||||||||||||||||||||||
Sbjct 1491940
ATGACCATTCTTCGACTTGTAGTAGTAACCGCGCGGCGCCTGCCGTAACGGCCTTCCAAG
1491999
Query 61
TCGTCTCGTCAAGCGCCCTCGACAACACTCACCACAGTGTTGGAACGAGGGCT 113
|||||||||||||||||||||||||||||||||||||||||||| ||||||||
Sbjct 1492000
TCGTCTCGTCAAGCGCCCTCGACAACACTCACCACAGTGTTGGACCGAGGGCT 1492052

>Corynebacterium deserti GIMN1.010, complete genome
Sequence ID: CP009220.1 Length: 2972149
Range 1: 1264918 to 1265030
Score:196 bits(216), Expect:1e-48,
Identities:111/113(98%), Gaps:0/113(0%), Strand: Plus/Plus
Query 1
ATGACCATTATTCGACTTGTAGTAGTAACCGCGCGGCGCCTGCCGTAACGGCCTTCCAAG 60
|||||||||||||||||||||||||||||||| |||||||||||||||||||||| ||||
Sbjct 1264918
ATGACCATTATTCGACTTGTAGTAGTAACCGCACGGCGCCTGCCGTAACGGCCTTACAAG
1264977
Query 61
TCGTCTCGTCAAGCGCCCTCGACAACACTCACCACAGTGTTGGAACGAGGGCT 113
|||||||||||||||||||||||||||||||||||||||||||||||||||||
Sbjct 1264978
TCGTCTCGTCAAGCGCCCTCGACAACACTCACCACAGTGTTGGAACGAGGGCT 1265030

>Corynebacterium crudilactis strain JZ16 chromosome, complete genome
Sequence ID: CP015622.1 Length: 3047373
Range 1: 1292205 to 1292317
Score:191 bits(211), Expect:5e-47,
Identities:110/113(97%), Gaps:0/113(0%), Strand: Plus/Plus
Query 1
ATGACCATTATTCGACTTGTAGTAGTAACCGCGCGGCGCCTGCCGTAACGGCCTTCCAAG 60
||||||||||||||||||||| ||||||||||||||||||||||||||||||||||||||
Sbjct 1292205
ATGACCATTATTCGACTTGTACTAGTAACCGCGCGGCGCCTGCCGTAACGGCCTTCCAAG
1292264
Query 61
TCGTCTCGTCAAGCGCCCTCGACAACACTCACCACAGTGTTGGAACGAGGGCT 113
|||||||||||||||||||||||||||||||||| ||||||||| ||||||||
Sbjct 1292265
TCGTCTCGTCAAGCGCCCTCGACAACACTCACCATAGTGTTGGACCGAGGGCT 1292317

>Corynebacterium callunae DSM 20147, complete genome
Sequence ID: CP004354.1 Length: 2839551
Range 1: 1252789 to 1252901
Score:182 bits(201), Expect:3e-44,
Identities:108/113(96%), Gaps:0/113(0%), Strand: Plus/Plus
Query 1
ATGACCATTATTCGACTTGTAGTAGTAACCGCGCGGCGCCTGCCGTAACGGCCTTCCAAG 60
||||||||||||||||||||| |||||||| | |||||||||||||| |||||| |||||
Sbjct 1252789
ATGACCATTATTCGACTTGTACTAGTAACCCCACGGCGCCTGCCGTAGCGGCCTACCAAG
1252848
Query 61
TCGTCTCGTCAAGCGCCCTCGACAACACTCACCACAGTGTTGGAACGAGGGCT 113
|||||||||||||||||||||||||||||||||||||||||||||||||||||
Sbjct 1252849
TCGTCTCGTCAAGCGCCCTCGACAACACTCACCACAGTGTTGGAACGAGGGCT 1252901

>Corynebacterium faecale strain DSM 45971 chromosome, complete genome
Sequence ID: CP047204.1 Length: 2987260
Range 1: 1295438 to 1295552
Score:156 bits(172), Expect:1e-36,
Identities:104/115(90%), Gaps:2/115(1%), Strand: Plus/Plus
Query 1
ATGACCATTATTCGACTTGTAGTAGTAACCGCGCGGCGCCTGCCGTAACGGCCTTC--CA 58
||||||||||||||||||||| ||||| | ||||||||| ||||||||||||||| ||
Sbjct 1295438
ATGACCATTATTCGACTTGTACTAGTAGCTGCGCGGCGCGTGCCGTAACGGCCTTTTACA
1295497
Query 59
AGTCGTCTCGTCAAGCGCCCTCGACAACACTCACCACAGTGTTGGAACGAGGGCT 113
|||||||||||||||||||||||||| |||||||||||||| || | ||||||||
Sbjct 1295498
AGTCGTCTCGTCAAGCGCCCTCGACAGCACTCACCACAGTGCTGAATCGAGGGCT 1295552

>Corynebacterium occultum strain 2039 chromosome, complete genome
Sequence ID: CP046455.1 Length: 3109186
Range 1: 1289608 to 1289719
Score:129 bits(142), Expect:1e-28,
Identities:97/113(86%), Gaps:1/113(0%), Strand: Plus/Plus

```
Query  1
ATGACCATTATTCGACTTGTAGTAGTAACCGCGCGGCGCCTGCCGTAACGGCCTTCCAAG  60
|||| |||||||||||||||||  ||| ||||||||||||||||||||||| ||| ||||
Sbjct  1289608
ATGATCATTATTCGACTTGTAGACGTACCCGCGCGGCGCCTGCCGTAACGG-CTTACAAG
1289666
Query  61
TCGTCTCGTCAAGCGCCCTCGACAACACTCACCACAGTGTTGGAACGAGGGCT  113
||||  || ||||||||||||||| ||| ||||||||||   |  ||||||||
Sbjct  1289667
TCGTACCGACAAGCGCCCTCGACAGCACCCACCACAGTGGCTGTTCGAGGGCT  1289719
```

>Corynebacterium vitaeruminis DSM 20294, complete genome
Sequence ID: CP004353.1 Length: 2931780
Range 1: 1319708 to 1319797
Score:106 bits(117), Expect:2e-21,
Identities:78/90(87%), Gaps:2/90(2%), Strand: Plus/Plus

```
Query  1
ATGACCATTATTCGACTTGTAGTAGTAACCGCGCGGCGCCTGCCGTAACGGCCTTC--CA  58
|||| ||||||||||||||| ||| || ||||||||||||||||||||||||   |  ||
Sbjct  1319708
ATGAACATTATTCGACTTGTGGTACTACCCGCGCGGCGCCTGCCGTAACGGCTCACCTCA
1319767
Query  59       AGTCGTCTCGTCAAGCGCCCTCGACAACAC  88
                |||||| |||||||||||||||| || |||
Sbjct  1319768  AGTCGTGTCGTCAAGCGCCCTCGTCAGCAC  1319797
```

>Corynebacterium hindlerae strain NML 93-0612 chromosome
Sequence ID: CP059833.1 Length: 2680694
Range 1: 2614900 to 2615007
Score:106 bits(117), Expect:2e-21,
Identities:91/109(83%), Gaps:4/109(3%), Strand: Plus/Plus

```
Query  6
CATTATTCGACTTGTAGTAGTAACCGCGCGGCGCCTGCCGTAACGGC-CTTCCAAGTCGT  64
|||||||||||||||| | |||    | |||||| ||||||| || | | | ||||||||
Sbjct  2614900
CATTATTCGACTTGTAATCGTACGTTCACGGCGCTTGCCGTAGCGACGCCTACAAGTCGT
2614959
Query  65
CTC--GTCAAGCGCCCTCGACAACACTCACCACAGTGTTGGAACGAGGG  111
| |  ||||||||||||||||||||| |||||||||||||| |||||||
Sbjct  2614960
CCCCAGTCAAGCGCCCTCGACAACACCCACCACAGTGTTGG-ACGAGGG  2615007
```

>Corynebacterium sp. SCR221107 chromosome, complete genome
Sequence ID: CP115670.1 Length: 3043024

Range 1: 1362659 to 1362768
Score:105 bits(116), Expect:2e-21,
Identities:93/113(82%), Gaps:5/113(4%), Strand: Plus/Plus
Query 1
ATGACCATTATTCGACTTGTAGTAGTAACCGCGCGGCGCCTGCCGTAACGGCCTTCC--A 58
|||| ||||||||||||||| ||| || | |||||||||||||||||||||| || |
Sbjct 1362659
ATGAACATTATTCGACTTGTGGTACTACCTGCGCGGCGCCTGCCGTAACGGCTCACCATA
1362718
Query 59
AGTCGTCTCGTCAAGCGCCCTCGACAACACTCACCACAGTGTTGGAACGAGGG 111
|||||| |||||||||||||||| || ||| ||| |||| |||||||||
Sbjct 1362719
AGTCGTGTCGTCAAGCGCCCTCGTCAGCAC-CAC--AAGTGCCTGAACGAGGG 1362768

>Corynebacterium humireducens NBRC 106098 = DSM 45392, complete genome
Sequence ID: CP005286.1 Length: 2681312
Range 1: 1155059 to 1155169
Score:105 bits(116), Expect:2e-21,
Identities:95/115(83%), Gaps:6/115(5%), Strand: Plus/Plus
Query 1
ATGACCATTATTCGACTTGTAGTAGTAACCGCGCGGCGCCTGCCGTAACG-GCCTTCCAA 59
|||| || ||||||||||| |||| | |||||||||||||||||||| ||| || |
Sbjct 1155059
ATGATCACCATTCGACTTGTGGTAGCA---GCGCGGCGCCTGCCGTAACGACCCTACCCA
1155115
Query 60
GTCGTCTCGTCAAGCGCCCTCGACAACACTCACCA-CAGTGTTGGAACGAGGGCT 113
||||||||| ||||||||||||||| ||| ||||| ||||| | | ||| |||||
Sbjct 1155116
GTCGTCTCGACAAGCGCCCTCGACAGCACCCACCATCAGTGCT-GTACGGGGGCT 1155169

>Corynebacterium halotolerans YIM 70093 = DSM 44683, complete genome
Sequence ID: CP003697.1 Length: 3135752
Range 1: 1402578 to 1402683
Score:100 bits(110), Expect:7e-20,
Identities:88/107(82%), Gaps:6/107(5%), Strand: Plus/Plus
Query 1
ATGACCATTATTCGACTTGTAGTAGTAACCGCGCGGCGCCTGCCGTAACGGCCT----TC 56
|||| || ||||||||||| |||||| | || ||||||||||||| |||| || ||
Sbjct 1402578
ATGATCAACATTCGACTTGTGGTAGTAGCTGCACGGCGCCTGCCGTGACGGTCTGACCTC
1402637
Query 57
CAAGTCGTCTCGTCAAGCGCCCTCGACAACACTCA-CCACAGTGTTG 102
| |||||||||| ||||||||||||||| ||| || |||||||| ||
Sbjct 1402638
C-AGTCGTCTCGACAAGCGCCCTCGACAGCACCCACCCACAGTGCTG 1402683

>Corynebacterium ulcerans strain MRi49 chromosome, complete genome

```
Sequence ID: CP046863.1 Length: 2527244
Range 1: 1082220 to 1082308
Score:97.8 bits(107), Expect:8e-19,
Identities:79/92(86%),  Gaps:3/92(3%), Strand: Plus/Plus
Query  1
ATGACCATTATTCGACTTGTAGTAGTAACCGCGCGGCGCCTGCCGTAACGGCCTTCCAAG  60
|||| ||||||||||||||||||  || ||| |||||||||||||||||||||  |||||
Sbjct  1082220
ATGAACATTATTCGACTTGTAGTGCTATCCGAGCGGCGCCTGCCGTAACGGCC-ACCAAG
1082278
Query  61       TCGTCTCGTCAAGCGCCCTCGACAACACTCAC  92
                ||||  ||| ||||||||||| || || ||||
Sbjct  1082279  TCGTAACGT-AAGCGCCCTCGCCAGCA-TCAC  1082308

>Corynebacterium silvaticum strain PO100/5 chromosome, complete
genome
Sequence ID: CP021417.2 Length: 2572864
Range 1: 1101124 to 1101212
Score:97.8 bits(107), Expect:8e-19,
Identities:79/92(86%),  Gaps:3/92(3%), Strand: Plus/Plus
Query  1
ATGACCATTATTCGACTTGTAGTAGTAACCGCGCGGCGCCTGCCGTAACGGCCTTCCAAG  60
|||| ||||||||||||||||||  || ||| |||||||||||||||||||||  |||||
Sbjct  1101124
ATGAACATTATTCGACTTGTAGTGCTATCCGAGCGGCGCCTGCCGTAACGGCC-ACCAAG
1101182

Query  61       TCGTCTCGTCAAGCGCCCTCGACAACACTCAC  92
                ||||  ||| ||||||||||| || || ||||
Sbjct  1101183  TCGTAACGT-AAGCGCCCTCGCCAGCA-TCAC  1101212

>Corynebacterium durum strain DSM 45333 chromosome, complete
genome
Sequence ID: CP047200.1 Length: 2786863
Range 1: 1143674 to 1143769
Score:92.4 bits(101), Expect:3e-17,
Identities:80/97(82%),  Gaps:2/97(2%), Strand: Plus/Plus
Query  1
ATGACCATTATTCGACTTGTAGTAGTAACCGCGCGGCGCCTGCCGTAACGGCCTTCCAAG  60
||||||||||||||||||||| | |||| | | |||||| |||||||||||   | ||||
Sbjct  1143674
ATGACCATTATTCGACTTGTACTCGTAAGCCCTCGGCGCTTGCCGTAACGGTTATACAAG
1143733
Query  61       TCGTCTCGTCAAGCGCCCTCGACAACA-CTCACCACA  96
                |||| ||||| ||||||||||  | || | |||||||
Sbjct  1143734  TCGTTTCGTC-AGCGCCCTCGGAAGCACCACACCACA  1143769

>Corynebacterium pseudotuberculosis strain PA09 chromosome
Sequence ID: CP054555.1 Length: 2338000
Range 1: 946805 to 946888
Score:90.6 bits(99), Expect:1e-16,
Identities:73/86(85%),  Gaps:2/86(2%), Strand: Plus/Plus
```

```
Query  2
TGACCATTATTCGACTTGTAGTAGTAACCGCGCGGCGCCTGCCGTAACGGCCTTCCAAGT  61
||| ||||||||||||||||||  || ||| |||||||||||||| ||||| | |||||
Sbjct  946805
TGAACATTATTCGACTTGTAGTGCTACCCGAGCGGCGCCTGCCGTAGCGGCCAT-CAAGT
946863
Query  62       CGTCTCGTCAAGCGCCCTCGACAACA  87
                |||  ||| ||||||||||| || ||
Sbjct  946864  CGTAACGT-AAGCGCCCTCGCCAGCA  946888

>Corynebacterium poyangense strain 4H37-19 chromosome, complete
genome
Sequence ID: CP046884.1 Length: 2617997
Range 1: 1094941 to 1095045
Score:86.0 bits(94), Expect:1e-15,
Identities:82/105(78%),  Gaps:4/105(3%), Strand: Plus/Plus
Query  1
ATGACCATTATTCGACTTGTAGTAGTAACCGCGCGGCGCCTGCCGTAACGGCCTT----C  56
||||| |||||||||||||||  | | | | | |||||||||||||| |||| ||
Sbjct  1094941
ATGACAATTATTCGACTTGTACGACTTAGCACTCGGCGCCTGCCGTAGCGGCTTTGTCAG
1095000

Query  57
CAAGTCGTCTCGTCAAGCGCCCTCGACAACACTCACCACAGTGTT  101
| ||||||| |||||||||||||| |  ||||||  | |||||||
Sbjct  1095001
CGAGTCGTCCCGTCAAGCGCCCTCAAATACACTCCACCCAGTGTT  1095045

>Corynebacterium diphtheriae strain CN2000 chromosome
Sequence ID: CP039522.1 Length: 2530683
Range 1: 1095415 to 1095501
Score:83.3 bits(91), Expect:2e-14,
Identities:73/89(82%),  Gaps:3/89(3%), Strand: Plus/Plus
Query  1
ATGACCATTATTCGACTTGTAGTAGTAACCGCGCGGCGCCTGCCGTAACGGCCTTCCAAG  60
|||| ||||||||||||||||||  | ||| | |||||| ||||||| ||||  | ||||
Sbjct  1095415
ATGAACATTATTCGACTTGTAGTGATTACCACTCGGCGCTTGCCGTAGCGGC--TACAAG
1095472
Query  61       TCGTCT-CGTCAAGCGCCCTCGACAACAC  88
                |||| | ||  |||||||||||||| |||
Sbjct  1095473  TCGTTTCCGAAAAGCGCCCTCGACAGCAC  1095501

>Corynebacterium gerontici strain W8 chromosome, complete genome
Sequence ID: CP033897.1 Length: 2263210
Range 1: 1005442 to 1005527
Score:82.4 bits(90), Expect:2e-14,
Identities:71/87(82%),  Gaps:1/87(1%), Strand: Plus/Plus
Query  2
TGACCATTATTCGACTTGTAGTAGTAACCGCGCGGCGCCTGCCGTAACGGCCTTCCAAGT  61
||| |||| |||||||||||||| | ||||  |||||||||||||| ||||| | |||||
```

```
Sbjct  1005442
TGAACATTCTTCGACTTGTAGTAATTACCGAACGGCGCCTGCCGTAGCGGCC-TGCAAGT
1005500
Query  62       CGTCTCGTCAAGCGCCCTCGACAACAC  88
                |||  || ||  |||||||| || |||
Sbjct  1005501  CGTAACGCCAGACGCCCTCGCCAGCAC  1005527

>Corynebacterium frankenforstense DSM 45800 strain ST18,
complete genome
Sequence ID: CP009247.1 Length: 2604152
Range 1: 1495561 to 1495637
Score:68.9 bits(75), Expect:4e-10,
Identities:62/77(81%),  Gaps:1/77(1%), Strand: Plus/Minus
Query  6
CATTATTCGACTTGTAGTAGTAACCGCGCGGCGCCTGCCGTAACGGCCTTCCAAGTCG-T  64
||||||| | |||||| | | |||||| |||||| ||||||||||| | | |||||||
Sbjct  1495637
CATTATTTGGCTTGTACTCGGAACCGCACGGCGCGTGCCGTAACGGACGTACAAGTCGAG
1495578
Query  65       CTCGTCAAGCGCCCTCG  81
                |||| | ||||||| ||
Sbjct  1495577  CTCGGCTAGCGCCCCCG  1495561
```
